# Water extract of *Rumex crispus* prevents bone loss by inhibiting osteoclastogenesis and inducing osteoblast mineralization

**DOI:** 10.1186/s12906-017-1986-7

**Published:** 2017-10-26

**Authors:** Ki-Shuk Shim, Bohyoung Lee, Jin Yeul Ma

**Affiliations:** KM Application Center, Korea Institute of Oriental Medicine, 70 Chemdan-ro, Dong-gu, 41062, Daegu, 701-300 Republic of Korea

**Keywords:** *Rumex crispus*, Bone loss, Osteoblast, Osteoclast, Runx2, Nuclear factor of activated T cells cytoplasmic 1

## Abstract

**Background:**

*Rumex crispus* root has traditionally been used in Asian medicine for the treatment of hemorrhage and dermatolosis. The aim of this study was to explore the pharmaceutical effects of water extract of *Rumex crispus* (WERC) on osteoblast and osteoclast differentiation. We also studied the effect of WERC on the receptor activator of nuclear factor kappa-B ligand (RANKL)-induced trabecular bone destruction mice model.

**Methods:**

High performance liquid chromatography analysis was used to identify three compounds (emodin, chrysophanol, and physcion) of WERC. The in vivo effect of WERC was examined using an administration of WERC or vehicle on the ICR mice with bone loss induced by intraperitoneal RANKL injection on day 0 and 1. All mice were sacrificed by cervical dislocation at day 7 and the femurs of mice were isolated for soft X-ray and Micro-CT analysis. The in vitro effect of WERC on osteoblast mineralization or osteoclast differentiation was examined by alizarin red S staining or by tartrate-resistant acid phosphatase staining and assay. To determine the transcription level of osteoblast or osteoclast-specific genes, real-time quantitative polymerase chain reaction was used. Western blot analysis was performed to study the effect of WERC on mitogen-activated protein kinases (MAPK) or nuclear factor-κB (NF-κB) signaling molecules.

**Results:**

The presence of three compounds in WERC was determined. WERC significantly suppressed RANKL-induced trabecular bone loss by preventing microstructural deterioration. In vitro, WERC increased osteoblast mineralization by enhancing the transcription of runt-related transcription factor 2 and its transcriptional coactivators, and by stimulating extracellular signal–regulated kinase phosphorylation. Furthermore, WERC significantly inhibited osteoclast differentiation by suppressing the activation of the RANKL signalings (MAPK and NF-κB) and the increasing inhibitory factors of nuclear factor of activated T cells cytoplasmic 1.

**Conclusion:**

This study showed that WERC could protect against osteoporosis and suggested that the possible mechanism of WERC might be related to increased osteoblast differentiation by activating Runx2 signaling and inhibition of osteoclast differentiation by suppression of RANKL signaling.

**Electronic supplementary material:**

The online version of this article (10.1186/s12906-017-1986-7) contains supplementary material, which is available to authorized users.

## Background

Bone loss in osteoporosis is a result of an imbalance in bone remodeling from an increased rate of osteoclast bone resorption that exceeds osteoblast bone formation. It is highly related to an increased incidence and recurrence of fractures in adults, which results in decreased quality of life and increased hospitalization and social health burden [1]. To develop treatment and prevention methods for osteoporosis, regulatory mechanisms of differentiation and/or activation of osteoclasts and osteoblasts have been intensively investigated. Osteoclasts are generated from bone marrow macrophages in the presence of key cytokines, macrophage-colony stimulating factor (M-CSF), and receptor activator of nuclear factor kappa-B ligand (RANKL) [2–4]. In the RANK signaling axis, the RANKL-induced nuclear factor of activated T cells cytoplasmic 1 (NFATc1) is activated through key signaling pathways including mitogen-activated protein kinases (MAPK) or NF-κB [5]. Osteogenic cytokines, such as bone morphogenetic protein (BMP), mainly differentiate mesenchymal stem cells into osteoblasts [6, 7]. This induces runt-related transcription factor 2 (Runx2) activation in cooperation with other transcription factors, including osterix, activating transcription factor 4 (ATF-4), NFATc1, special AT-rich sequence-binding protein 2 (SATB2), or activator protein-1 (AP-1) via MAPK signal pathways to regulate the transcription of osteoblast-specific genes during osteoblast differentiation [8–10].

*Rumex crispus* root has been traditionally used to treat hemoptysis, scabies, hematochezia, and neurodermatitis in Asian medicine. Its pharmaceutical ability to scavenge free radicals, inhibit proliferation and induce apoptosis of cancer cells, and to suppress plant pathogenic fungi has been recently studied [11–13]. In terms of the effect on bone cell differentiation or proliferation, an ethanol extract of *R. crispus* root significantly stimulates alkaline phosphatase (ALP) activity, but partially increases bone nodule formation in osteosarcoma MG-63 cells [14]. However, the molecular mechanism controlling how *R. crispus* induces and regulates osteoblast differentiation has not been fully investigated. In addition, there have been no studies addressing the effect of *R. crispus* on osteoclast differentiation and its related molecular mechanisms. Thus, we explored the effect of water extract of *R. crispus* (WERC) on osteoblast differentiation using primary osteoblasts and on osteoclast differentiation using bone marrow macrophages (BMMs). In addition, we also explored the effect of WERC on Runx signaling in osteoblasts and RANKL signaling in osteoclasts to elucidate the molecular action mechanism of WERC. RANKL-induced bone loss model is the simple and fast osteoporosis model that could be used to evaluate drug candidates for osteoporosis [15]. To explore the in vivo effects of *R. crispus* on bone destruction, we evaluated the activity of WERC on RANKL-induced bone destruction model and characterized the trabecular bone microstructure by analyzing bone parameters.

## Methods

### Preparation of WERC

To prepare water extract of *R. crispus* (Yeongcheon herb, Yeongcheon, Korea), 50 g of dried *R. crispus* (Specimen No. W292) was soaked in distilled water (1 L) and then extracted by boiling for 3 h [16]. Following filtration, the water extract was evaporated under reduced pressure for lyophilization. The lyophilized powder was re-suspended in distilled water, centrifuged at 10,000×*g* for 15 min, and filtered through a 0.2-μm sterile filter to prepare WERC.

### High performance liquid chromatography (HPLC) analysis of WERC

The HPLC system (Dionex Co., Sunnyvale, CA, USA) used in this study consisted of an ultimate 3000 series A binary pump, an auto-sampler, a column oven and a diode array UV/VIS detector (DAD). Data acquisition was performed using the Dionex Chromelon software. Chromatographic separation was achieved on a Thermo Acclaim C_18_ column (Thermo Fisher Scientific Inc., Waltham, MA, USA) using phosphoric acid water (0.1%, *v*/v), solvent A and acetonitrile, and solvent B as a mobile phase at a flow rate of 1 mL/min. The HPLC elution conditions were optimized as follows: (0–8) min, 15–13% B; (8–12) min, 13–35% B; (12–22) min, 35–75% B; (22–32) min, 75% B. The column oven and auto-sampler injection volume were set to 30 °C and 10 μL, respectively. The detected wavelength was set at 280 nm and the total run time was 73 min. WERC (12.5 mg) was immersed in 1 mL of 100% methanol and extracted using ultra sonication for 30 min. The standard stock solutions were prepared by dissolving accurately weighed compounds in 100% methanol (1 mg/mL). All working solutions were filtered through 0.2-mm syringe membrane filters from Whatman Ltd. (Maidstone, UK) before injection into the HPLC-DAD system.

### Bone loss model

Animal experiments were handled in accordance with the guidelines of the Korea Food and Drug Administration Guide for the Care and Use of Laboratory Animals. The experiment plan was reviewed and approved by the Institutional Animal Care and Use Committee (IACUC) at the Korea Institute of Oriental Medicine (approval number; 12–121). Specific-pathogen-free ICR mice (7-week-old males, 32.16 ± 4.1 g, total 18 mice) were obtained (Samtako Bio Inc., Korea) and acclimatized for 1 week. All mice were housed at 22 ± 1 °C and 55 ± 10% humidity on a 12-h light/dark cycle and received pathogen-free water and food for maintenance. 6 mice per group were taken according to a stochastic averaging principle. The initial body weight and health status of the mice were no significant difference among the groups in this study. The mice were randomly divided into two groups: phosphate buffered saline (PBS)-injected control group, RANKL (1 mg/kg of body weight)-injected group. After intraperitoneally injection with RANKL (1 mg/kg of body weight) or PBS on days 0 and 1, the RANKL-injected mice were orally administered vehicle (distilled water) or WERC (0.25 g/kg of body weight) twice daily for five consecutive days [15]. At day 7, all mice were sacrificed by cervical dislocation and the femurs of mice were isolated for soft X-ray and Micro-CT analysis. Micro-CT scanning of the distal femur was carried out on the Quantum FX scanner system (PerkinElmer, Inc., MA, USA). Bone parameters including trabecular bone volume per tissue volume (BV/TV), trabecular thickness (Tb.Th), trabecular number (Tb.N), and trabecular separation (Tb.Sp) were measured between 0.54 and 1.46 mm distal to the growth plate.

### Cell culture and enzyme assay

Animal experiments were approved by the IACUC at the Korea Institute of Oriental Medicine (approval numbers, 14–083 and 14–085). Calvarial osteoblasts isolated from calvariae of newborn mice (postnatal day 2) by five time sequential enzyme digestion (0.1% collagenase and 0.2% dispase) and BMMs were generated from mouse bone marrow cells as previously reported [17]. Cell viability was determined using the Cell Counting Kit-8 (Dojindo, USA) after incubation with WERC for 2 days. Osteoblasts (1 × 10^5^ cells/well, 24 well plate) were cultured in growth medium [GM, DMEM supplemented with 10% fetal bovine serum (FBS)] or differentiation medium (DM, α-MEM medium supplemented with 10% FBS, 50 μg/mL ascorbic acid, and 10 mM β-glycerophosphate) for 7–15 d. To differentiate osteoclasts, BMMs (1 × 10^4^ cells/well in a 96-well plate) were cultured with M-CSF and RANKL for 4 d. The ALP activity assay, ALP staining, and alizarin red S quantification were used to evaluate osteoblast differentiation. The tartrate-resistant acid phosphatase (TRAP) activity assay and staining were used for osteoclast differentiation as previously described [18, 19]. The representative image of cells was taken under the microscope.

### Real-time quantitative polymerase chain reaction (qRT-PCR)

Osteoblasts (1 × 10^5^ cells/well, 48-well plate) or osteoclasts (4 × 10^5^ cells/well, 6-well plate) were treated with vehicle (distilled water) or the indicated concentration of WERC. Total RNA (1 μg) was used for cDNA synthesis with a cDNA synthesis kit (Bioneer Inc., Daejeon, Korea). qPCR analysis was performed with the CFX96 Touch Real-Time PCR System (Bio-Rad, Hercules, CA, USA) using AccuPower GreenStar qPCR Master mix as recommended by the manufacturer (Bioneer). The PCR conditions involved activation at 95°C for 5 min and 40 cycles of amplification at 94°C for 20 s and at 60°C for 40 s. The data were analyzed using the CFX manager software (Version 3.1). Hypoxanthine phosphoribosyltransferase was used as an internal control.

### Western blot analysis

BMMs (4 × 10^5^ cells/well, 6-well plate) or primary osteoblasts (1 × 10^5^ cells/well, 48-well plate) were treated with vehicle (distilled water) or WERC for indicated time periods. BMMs were incubated with RANKL and M-CSF for osteoclast differentiation. The cells were lysed in RIPA buffer containing protease and phosphatase inhibitor as previously reported [16]. Total protein lysates (30 μg) were subjected to western blot analysis using specific primary antibodies and secondary antibodies. All antibodies were from Cell Signaling Technology (USA), with the exception of c-Fos and NFATc1 (Santa Cruz Biotechnology, USA). Chemiluminescent signals were detected with Pierce ECL Western Blotting Substrate (Bio-Rad). The intensities of the bands were analyzed using Image Lab software (version 5.2.1, Bio-Rad).

### Statistical analysis

The difference in bone parameters between groups (6 mice per group) were analyzed using one-way analysis of variance followed by Dunnett’s test for multiple comparisons. Student’s *t*-test was performed to evaluate the significance in the enzyme activity assay and mRNA levels of genes. Data were presented as means ± standard deviation of three independent experiments, with the exception of the animal experiment. *p* < 0.05 was considered statistically significant.

## Results

### HPLC analysis of WERC

**Fig. 1:**
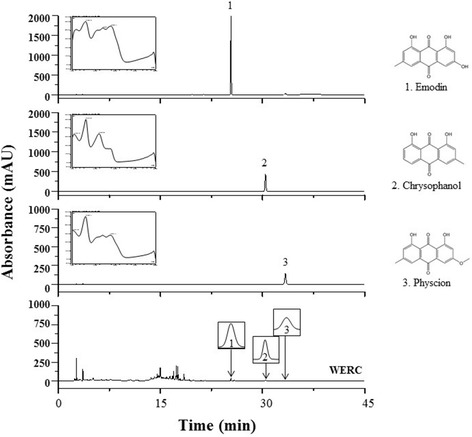
HPLC-DAD chromatogram of WERC and three standard components (emodin, chrysophanol, and physcion) at wavelengths of 280 nm

A typical HPLC profile is illustrated in Fig. 1 where peaks 1, 2, and 3 were identified as emodin, chrysophanol, and physcion, respectively, by comparing their retention times and ultraviolet (UV) spectral data with the standard compounds. The detection wavelength was selected according to the maximum absorption of the compounds in the UV spectra obtained with a diode array detector coupled to the HPLC system. The retention times of emodin (25.37 min), chrysphanol (30.44 min), and physcion (33.37 min) are indicated in the chromatogram.

### WERC suppressed RANKL-induced trabecular bone loss in mice

**Fig. 2:**
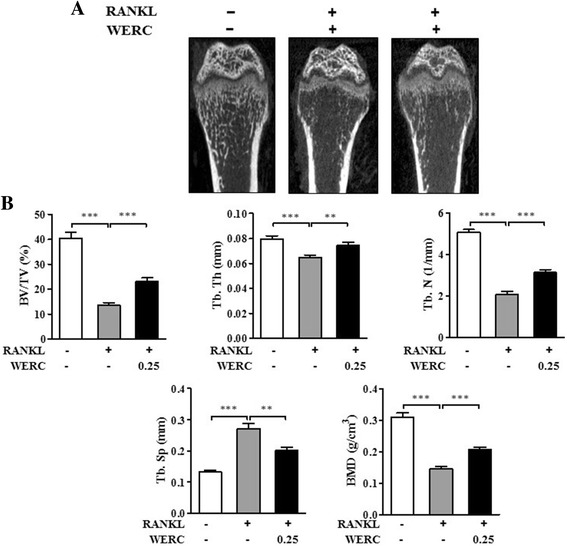
(**a**) Tibia images were obtained by X-ray analysis at the proximal tibia below the growth plate as described in the “Methods”. (**b**) Trabecular bone volume/tissue volume (BV/TV), trabecular thickness (Tb.Th), trabecular number (Tb.N), trabecular separation (Tb.Sp), and bone mineral density (BMD) were quantified at the proximal tibia using micro-CT analysis

We first investigated the in vivo effect of WERC in an RANKL-induced bone loss mouse model, known as the acute trabecular bone loss model. As shown in Fig. 2a, RANKL injection markedly abolished the trabecular structure at the metaphysis of the femur compared to vehicle treatment. However, oral administration of WERC (0.25 g/kg) for 5 d protected RANKL-induced loss of the trabecular structure. In agreement with the X-ray results, BV/TV, Tb.Th, Tb.N, and BMD in the WERC group were significantly increased, but Tb.Sp in in the WERC group was significantly decreased compared to the RANKL group (Fig. 2b), suggesting that WERC exerts its bone-protecting effect in an RANKL excess state.

### WERC stimulates osteoblast mineralization by increasing transcription factor expression and ERK phosphorylation

**Fig. 3:**
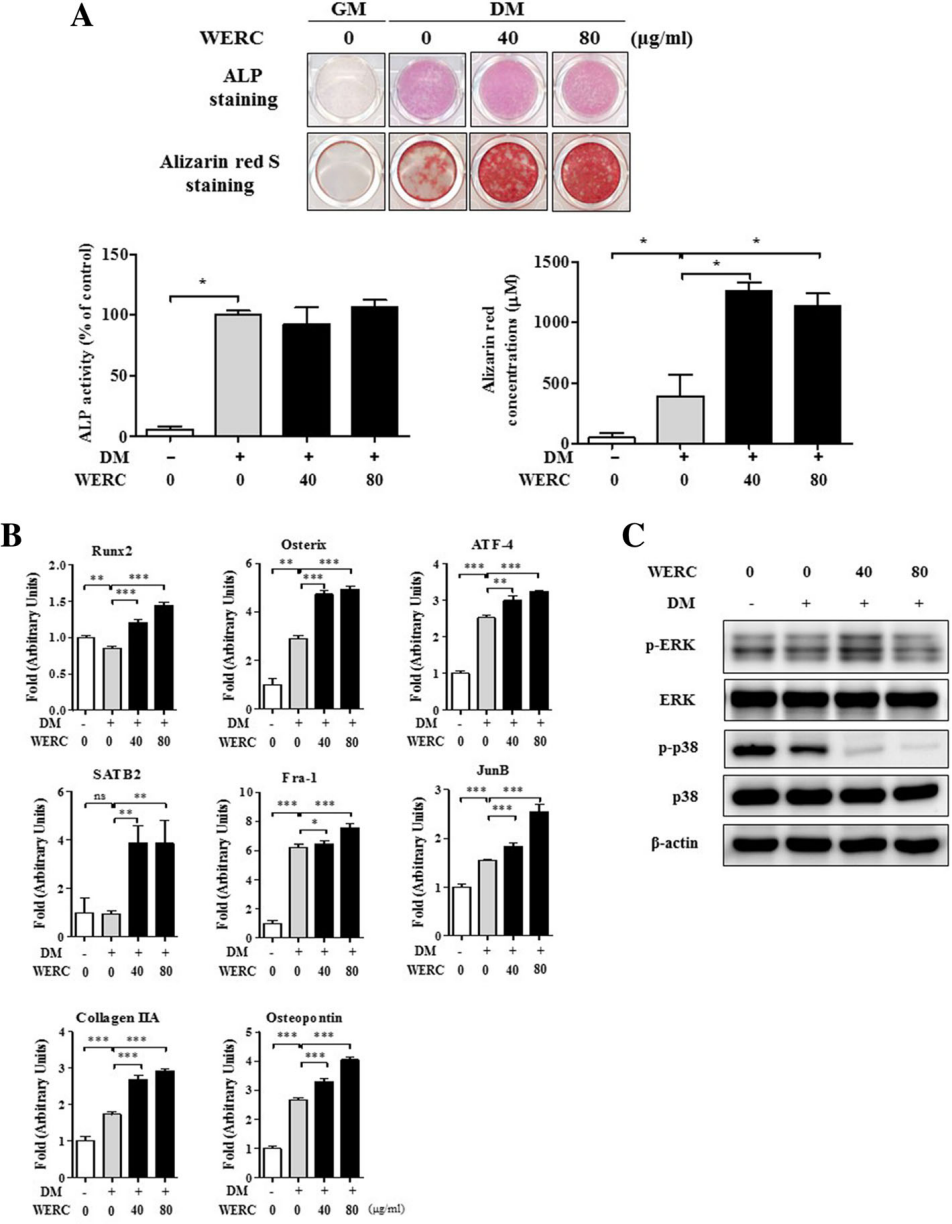
WERC stimulates osteoblast mineralization by increasing osteoblast gene expression and ERK signaling. Osteoblasts isolated from mouse calvariae were cultured in growth medium (GM, α-MEM) or differentiation medium (DM; 50 μg/mL ascorbic acid, and 10 mM β-glycerophosphate in α-MEM) in the presence of WERC (40 and 80 μg/mL). (**a**) Osteoblasts were subjected to ALP staining on day 5 (upper panel) or alizarin red S staining on day 10 (lower panel) (40 × magnification). Quantification of ALP activity was determined using the ALP activity assay. Quantification of mineralization was determined by measuring the amount of alizarin red S dye extracted from stained cells. (**b**) mRNA levels of Runx2, osterix, ATF-4, SATB2, Fra-1, JunB, collagen IIA, and osteopontin were determined by qRT-PCR on day 10. (**c**) After culturing osteoblasts in DM and WERC to 10 days, total cell lysate (30 μg) was extracted and subjected to western blot analysis with antibodies specific to ERK, p38, and β-actin. *p < 0.05, **p < 0.01, ***p < 0.001

Regarding the effect of bone cell differentiation or proliferation, an ethanol extract of *R. crispus* root partially stimulates ALP activity and bone nodule formation as well as proliferation in osteosarcoma MG-63 cells. Thus, we investigated the effect of WERC using an in vitro osteoblast culture system with primary osteoblast precursors isolated from calvariae of newborn mice, and staining with ALP and alizarin red S. As shown in Fig. 3a, differentiated osteoblasts were stained with ALP or alizarin red S at 7 and 15 d, respectively. The contents of ALP or alizarin red S staining increased 100–400-fold, whereas WERC (40–80 μg/mL) further increased the alizarin red S staining up to 3-fold compared to the vehicle.

Several signaling molecules and transcription factors play a pivotal role in regulating osteoblast differentiation. Using qRT-PCR, we found that WERC significantly increased the mRNA levels of transcription factor (Runx2, osterix, ATF-4, SATB2, Fra-1, and JunB) and osteoblast marker (collagen IIA and osteopontin) genes in a dose-dependent manner (Fig. 3b). Interestingly, WERC increased the levels of osterix, SATB2, JunB, and osteopontin to approximately 2-fold compared to the vehicle. To determine the upstream signaling molecules involved, we examined MAPK (ERK and p38) phosphorylation and found that WERC increased ERK phosphorylation, but decreased p38 phosphorylation (Fig. 3c). These results suggest that WERC stimulates osteoblast mineralization by upregulating key transcriptional factors and its related signaling molecules in late stages of osteoblast differentiation.

### WERC inhibits osteoclast differentiation by inhibiting RANKL axis

**Fig. 4:**
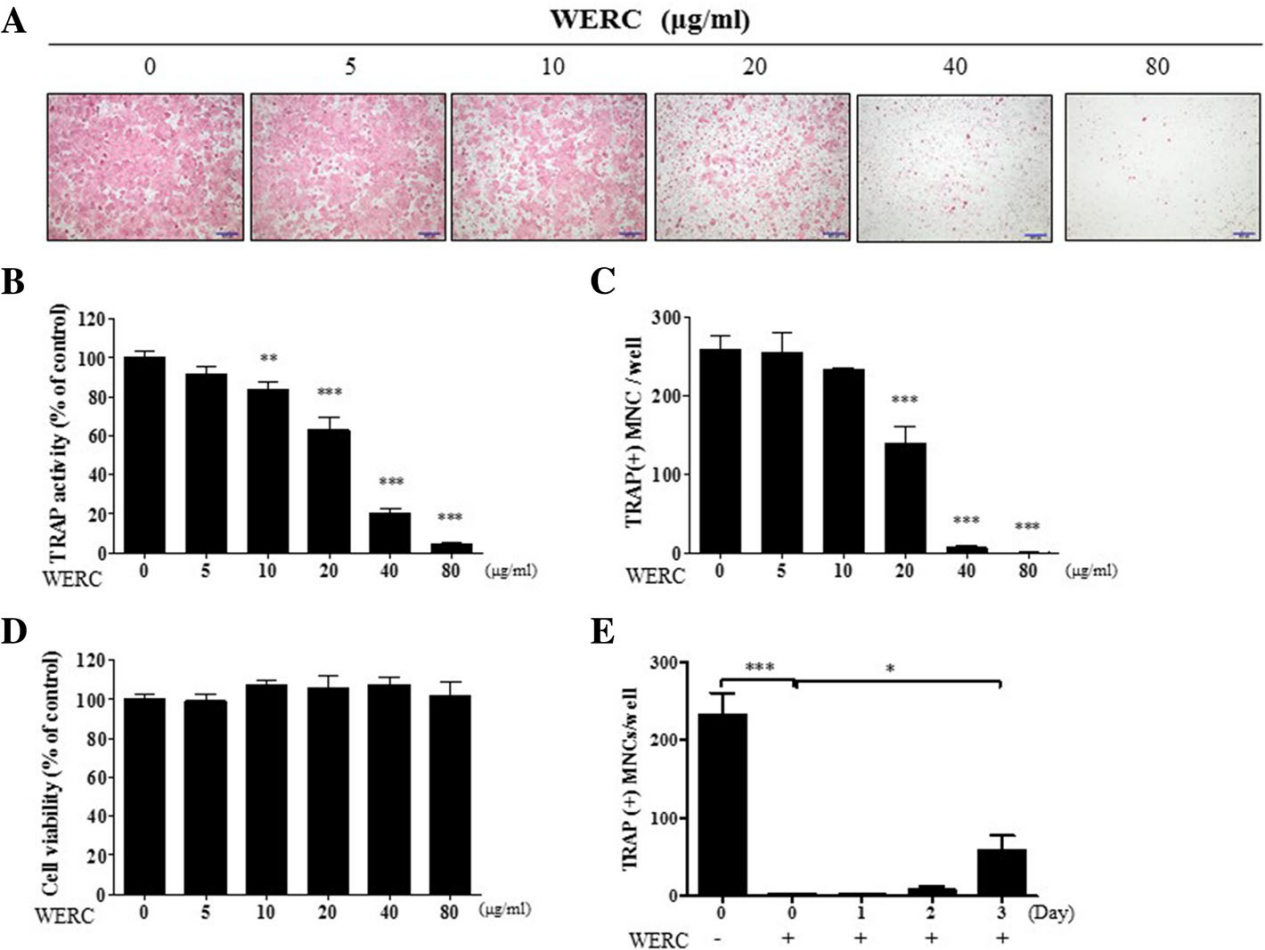
WERC suppresses osteoclast differentiation. BMMs were cultured in differentiation medium (DM) containing M-CSF and RANKL for 4 days with vehicle or WERC (10, 20, 40, 80, and 160 μg/mL). The cells cultured in DM were stained for (**a**) TRAP staining and were determined by the (**b**) TRAP activity assay on day 4 (100 × magnification). (**c**) TRAP-positive MNCs were counted on day 4. (**d**) The cells were cultured with WERC and M-CSF to examine cell viability on day 2. (**e**) The cells were cultured in DM in between additions of WERC (80 μg/mL) at the indicated days, and the osteoclast numbers were counted on day 4. *p < 0.05, **p < 0.01, ***p < 0.001

Since the inhibitory effect of WERC in the in vivo model could result from its activity in osteoclastogenesis, we determined the effect of WERC on RANKL-induced osteoclast differentiation of BMMs using TRAP staining and a TRAP activity assay. As shown in Fig. 4a–c, WERC significantly decreased TRAP-stained osteoclasts and TRAP activity in a dose-dependent manner without altering the viability of BMMs up to 80 μg/mL of WERC (Fig. 4d). When we counted the mononuclear cell (MNC) number at different days after WERC treatment, WERC almost completely suppressed MNC formation at day 0–2 while suppressing 80% of MNC formation on day 3 (Fig. 4e), suggesting that WERC mainly targets the early phase of osteoclast differentiation from BMMs to osteoclasts.

**Fig. 5:**
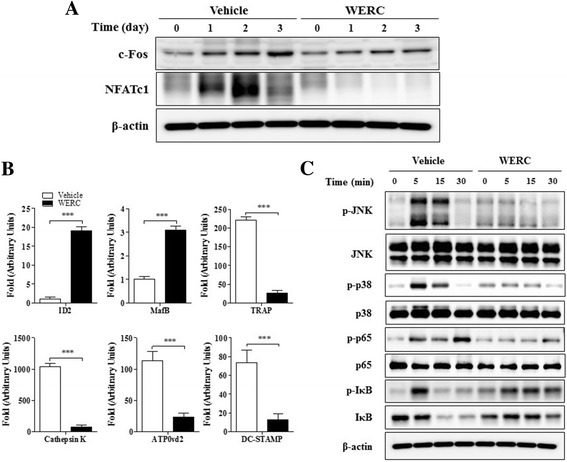
WERC inhibits c-Fos and NFATc1 expression in osteoclasts. BMMs were incubated with vehicle or WERC (80 μg/mL) for 4 days in the presence of M-CSF and RANKL. (**a**) Total cell lysate collected for each time point was subjected to western blot analysis with c-Fos or NFATc1 antibody. (**b**) mRNA levels of Id2, MafB, TRAP, cathepsin K, ATPv0d2, and DC-STAMP were analyzed by qRT-PCR on day 3. (**c**) BMMs pre-treated with vehicle or WERC (80 μg/mL) in the presence of M-CSF for 3 h were incubated with RANKL (100 ng/mL) for 0, 5, 15, and 30 min. Total cell lysate (30 μg) was subjected to western blot analysis with antibodies specific to JNK, p38, p65, and I-κB. ***p < 0.001 vs. vehicle

Next, we investigated the effect of WERC on osteoclast transcriptional factors and its related signaling molecules to elucidate the inhibitory mechanisms of WERC in osteoclastogenesis. We found that WERC completely abolished NFATc1 expression and significantly downregulated NFATc1-regulated osteoclast markers (TRAP, cathepsin K, ATPv0d2, and DC-STAMP) (Fig. 5b). In addition, WERC increased NFATc1-inhibitory transcription factor (ID2 and MafB), but decreased the expression of c-Fos, known as upstream factor of NFATc1 transcript (Fig. 5a and b). Furthermore, when we examined MAPK and NF-κB as a major signaling pathway in osteoclastogenesis, we found that WERC suppressed JNK, p38, and p65 phosphorylation but inhibited I-κB phosphorylation for degradation (Fig. 5c). These results suggest that WERC inhibits osteoclast differentiation by downregulating key transcription factors and its upstream signaling molecules in RANKL axis.

## Discussion

*Rumex crispus* roots have been used to treat hemorrhage and dermatolosis in Korean medicine. The potential osteogenic activity of *R. crispus* roots has been suggested, but the molecular mechanism of this activity remain unclear. Our results demonstrate that WERC stimulates osteoblast mineralization by enhancing osteogenic transcription factors and stimulating ERK signaling. Furthermore, WERC suppressed RANKL-induced osteoclast differentiation and trabecular bone loss in mouse.

We found that WERC enhances the mRNA levels of stimulatory transcription factors to induce osteogenesis. Runx2 is the master osteoblast factor that induces osterix expression and physically interacts with ATF-4 and SATB2, enhancing their transcriptional regulation of osteoblast marker genes [9, 20, 21]. Mice deficient in an AP-1 family member display decreased levels of several matrix proteins, such as osteocalcin, suggesting the key role of AP-1 in osteoblast differentiation [22, 23]. Among osteogenic signaling pathways, it is known that ERK and p38 regulate osteoblast markers in osteoblast differentiation differently. p38 mediates upregulation of ALP, osteocalcin, and type I collagen of C2C12 and/or human osteoblasts while ERK upregulates fibronectin and osteopontin in osteoblasts [24, 25]. In addition, p38 phosphorylation induces the activation of Runx2 via the TAK1/MEK3 pathway, but ERK1/2 directly mediates Runx2 phosphorylation and transcriptional activity at early stages of differentiation [26, 27]. We found that WERC increases ERK activation and transcription of key transcription factors with an increase of mineralization in late stages of osteoblast differentiation, but did not stimulate ALP activity and staining. Therefore, our findings suggest that WERC activity could occur at terminal stages of the osteoblast differentiation process via ERK/Runx2 signaling.

We found that WERC significantly suppressed RANKL-induced trabecular bone loss in an in vivo model. Either stimulation of osteoclast differentiation or activation of pre-existing mature osteoclasts has been suggested to be the mechanism of bone loss in this model [15]. Consistent with in vivo activity of WERC, we first found that WERC markedly inhibited in vitro osteoclast differentiation by suppressing the MAPK/NF-κB/NFATc1 signaling axis. This suggests that the mechanism of WERC in the in vivo model results from its inhibitory activity on osteoclast differentiation although its effect on the activation of mature osteoclasts is needed to determine. For the inhibitory mechanism of osteoclast differentiation, WERC not only inhibited the key factors of the RANKL/NFATc1 axis, but also increased inhibitory factors of NFATc1 such as ID2 which regulates the initiation of differentiation. Furthermore, WERC markedly suppressed the fusion of pre-osteoclast as shown in the MNC formation results. Fusion is one of the key processes of differentiation to generate multi-nuclear giant osteoclast after an initiation of osteoclast differentiation. This suggests that the inhibitory activity of WERC on bone loss might originate from several components of WERC targeting different molecules involved in each process of osteoclast differentiation.

The chemical constituents of *R. crispus* root include various phenolic components, including anthraquinone, nepodin, chrysophanol, chrysophanein, emodin, and physcion [28, 29]. In this study, we identified the presence of anthraquinone compounds, such as emodin, physcion, and chrysophanol, in WERC by HPLC analysis. In addition, we found that these components (emodin, chrysophanol, and physcion) significantly inhibited RANKL-induced osteoclast differentiation, or that some of them (chrysophanol and physcion) enhanced osteoblast differentiation (Additional file 1: Figure S1) as reported in previous studies [30, 31]. Thus, it might suggest that WERC activity against osteoporotic bone loss could result from some active components including anthraquinone components in WERC by regulating bone cell differentiation.

In a previous study evaluating the pharmacological activity of *R. crispus* on osteoblast differentiation, an ethanol extract of *R. crispus* root and leaf had different osteogenic activity on osteosarcoma MG-63 cells [32]. *R. crispus* root had a stimulatory effect on ALP activity but had little effect on mineralization, which is opposite to the effect of *R. crispus* leaf on the cells. In contrast to this previous report, WERC significantly stimulated mineralization, but did not increase ALP activity or staining of primary osteoblasts. These discrepancies might be a result of differences in the cell types tested (primary osteoblasts vs. osteosarcoma cells), in the extraction protocol of *R. crispus* root (water extraction vs. 70% ethanol extraction), or in the osteogenic culture medium (α-MEM vs. DMEM containing dexamethasone).

## Conclusions

In conclusion, our studies demonstrated that WERC induces osteoblast differentiation by stimulating Runx2 signaling and related transcription factors. Moreover, WERC inhibited RANKL-induced osteoclast differentiation by suppressing the RANKL signaling axis, and prevents bone loss in a mouse model. This suggests that WERC could be used as a pharmacological candidate for traditional medicine in the prevention and treatment of osteoporosis.

## Additional file

Additional file 1: Figure S1. (A) BMMs were cultured with vehicle (dimethyl-sulfoxide) or the indicated compounds (emodin, chrysophanol, physcion) in the presence of M-CSF and RANKL for TRAP activity assay and staining (100 × magnification). Cell viability were examine on day 2. **p* < 0.05, ***p* < 0.01, ****p* < 0.001 vs vehicle. (B) Mouse osteoblast were cultured with vehicle or these compounds in growth medium (GM) or differentiation medium (DM) for 10 days. Osteoblast mineralization was evaluated by Alizarin red S staining (100 × magnification) and quantification of alizarin red S dye stained. **p* < 0.05, ***p* < 0.01 vs differentiation medium (DM). (JPEG 185 kb)

## Abbreviations

BMMs: bone marrow-derived macrophage cells
CT: computed tomography
CTX: C-terminal cross-linked telopeptide of type I collagen
HPLC: High performance liquid chromatography
JNK: c-Jun N-terminal kinase
M-CSF: Macrophage colony-stimulating factor
NFATc1: Nuclear factor of activated T-cells cytoplasmic 1
NF-κB: Nuclear factor kappa B
PBS: Phosphate-buffered saline
qPCR: Quantitative real-time polymerase chain reaction
RANKL: Receptor activator for nuclear factor-κB ligand
TRAP (+) MNCs: TRAP-positive multinucleated cells
TRAP: Tartrate-resistant acid phosphatase
WERC: water extract of *Rumex crispus*
α-MEM: Minimum Essential Medium Alpha modification
