# Supplementary figures and images for: Water extract of Rumex crispus prevents bone loss by inhibiting osteoclastogenesis and inducing osteoblast mineralization
Source: BMC Complement Altern Med. 2017 Oct 26;17:483. doi: 10.1186/s12906-017-1986-7 (PMC5657118; doi:10.1186/s12906-017-1986-7)

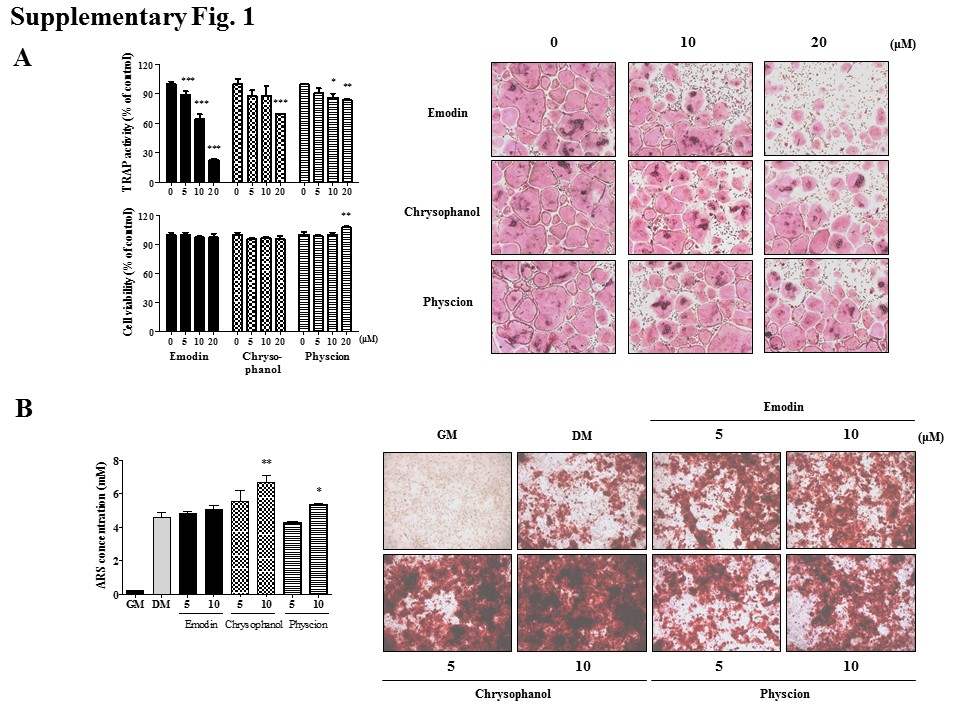

Supplement: Additional file 1: Figure S1. — (A) BMMs were cultured with vehicle (dimethyl-sulfoxide) or the indicated compounds (emodin, chrysophanol, physcion) in the presence of M-CSF and RANKL for TRAP activity assay and staining (100 × magnification). Cell viability were examine on day 2. *p < 0.05, **p < 0.01, ***p < 0.001 vs vehicle. (B) Mouse osteoblast were cultured with vehicle or these compounds in growth medium (GM) or differentiation medium (DM) for 10 days. Osteoblast mineralization was evaluated by Alizarin red S staining (100 × magnification) and quantification of alizarin red S dye stained. *p < 0.05, **p < 0.01 vs differentiation medium (DM). (JPEG 185 kb) [file 12906_2017_1986_MOESM1_ESM.jpg]
